# Leveraging International Influenza Surveillance Systems and Programs during the COVID-19 Pandemic

**DOI:** 10.3201/eid2813.212248

**Published:** 2022-12

**Authors:** Perrine Marcenac, Margaret McCarron, William Davis, Ledor S. Igboh, Joshua A. Mott, Kathryn E. Lafond, Weigong Zhou, Marjorie Sorrells, Myrna D. Charles, Philip Gould, Carmen Sofia Arriola, Vic Veguilla, Erica Guthrie, Vivien G. Dugan, Rebecca Kondor, Eric Gogstad, Timothy M. Uyeki, Sonja J. Olsen, Gideon O. Emukule, Siddhartha Saha, Carolyn Greene, Joseph S. Bresee, John Barnes, David E. Wentworth, Alicia M. Fry, Daniel B. Jernigan, Eduardo Azziz-Baumgartner

**Affiliations:** Centers for Disease Control and Prevention, Atlanta, Georgia, USA

**Keywords:** influenza, COVID-19, coronavirus disease, SARS-CoV-2, severe acute respiratory syndrome coronavirus 2, viruses, respiratory infections, zoonoses, vaccine-preventable diseases, epidemiologic surveillance, vaccine deployment

## Abstract

A network of global respiratory disease surveillance systems and partnerships has been built over decades as a direct response to the persistent threat of seasonal, zoonotic, and pandemic influenza. These efforts have been spearheaded by the World Health Organization, country ministries of health, the US Centers for Disease Control and Prevention, nongovernmental organizations, academic groups, and others. During the COVID-19 pandemic, the US Centers for Disease Control and Prevention worked closely with ministries of health in partner countries and the World Health Organization to leverage influenza surveillance systems and programs to respond to SARS-CoV-2 transmission. Countries used existing surveillance systems for severe acute respiratory infection and influenza-like illness, respiratory virus laboratory resources, pandemic influenza preparedness plans, and ongoing population-based influenza studies to track, study, and respond to SARS-CoV-2 infections. The incorporation of COVID-19 surveillance into existing influenza sentinel surveillance systems can support continued global surveillance for respiratory viruses with pandemic potential.

The persistent threat of influenza has spurred decades of work to build global surveillance, preparedness, and capacity to respond to seasonal, zoonotic, and pandemic influenza. Activities to support international laboratory and epidemiology capacity building for early detection and response to influenza and other respiratory viruses have been conducted through close collaboration between the World Health Organization (WHO), country ministries of health (MOH), other national health agencies such as the US Centers for Disease Control and Prevention (CDC), nongovernmental organizations (NGOs), academic research groups, and many others. These partnerships helped to prepare countries to respond to seasonal influenza outbreaks, the emergence of human infections with highly pathogenic avian influenza A(H5N1) virus starting in 2004, the 2009 influenza A(H1N1) pandemic (*1*), and the COVID-19 pandemic.

A central component of building global influenza surveillance capacity has been the Global Influenza Surveillance and Response System (GISRS). GISRS, established in 1952 by WHO to monitor circulating influenza virus strains to improve strain selection for seasonal and pandemic influenza vaccines, operates through a network of 148 National Influenza Centres (NICs), 7 Collaborating Centers (CCs), 4 Essential Regulatory Laboratories, and 13 H5 Reference Laboratories (*1*,*2*). Since 1956, the CDC Influenza Division, part of the National Center for Immunization and Respiratory Diseases, has served as a WHO CC for Surveillance, Epidemiology, and Control of Influenza as part of GISRS. In this role, the division has supported global expansion of year-round epidemiologic and virologic surveillance for rapid detection and characterization of seasonal influenza viruses, other respiratory viruses, and other viruses with pandemic potential (*3*). Starting in 2004, the Influenza Division developed an international program with the objective of increasing global contributions to GISRS through the establishment of new or expansion of existing national influenza surveillance systems. The division has provided technical and financial assistance to >120 partners in >70 countries to improve influenza prevention and control activities. The Influenza Division established and maintained 5-year cooperative agreements with partner countries and WHO Regional Offices to support influenza surveillance capacity building and pandemic preparedness activities. Moreover, Influenza Division staff have been posted in 17 overseas locations at various times, including at WHO headquarters and Regional Offices, to work closely with public health partners.

On March 11, 2020, WHO made the assessment that COVID-19, the disease caused by SARS-CoV-2 infection, was a pandemic (*4*). In this report, we describe global influenza surveillance systems, pandemic preparedness activities, and partnerships and how these helped the international and country-specific response to the COVID-19 pandemic, focusing on programs and activities supported by the CDC Influenza Division. We also present perspectives about how these programs can continue to support surveillance for influenza, SARS-CoV-2, and other respiratory viruses and bolster preparedness for respiratory viruses with epidemic and pandemic potential.

## Leveraging of Influenza Surveillance Infrastructure and Systems for SARS-CoV-2 Surveillance

The Influenza Division’s first international program activities in 2004 included supporting the MOH of 9 countries to build or expand their national influenza surveillance systems and pandemic preparedness. By 2018, CDC funding support for influenza surveillance reached >70 countries, in part because of the 2009 H1N1 pandemic response (*5*). Through these activities, WHO, MOH, the Influenza Division, and others at CDC, including the Division of Global Health Protection, Center for Global Health, have defined and standardized severe acute respiratory infection (SARI) and influenza-like illness (ILI) case definitions for respiratory disease surveillance. The Influenza Division assisted in the development of WHO’s global surveillance guidelines to standardize how influenza surveillance is conducted across countries and reported to FluNet as part of the GISRS platform (*6*). The division also supported countries as they established sentinel surveillance sites for the identification of persons with influenza or other respiratory virus infections. Along with partners, the Influenza Division provided courses on data analysis, data management, response procedures for respiratory outbreaks, and surveillance system evaluation, and conducted site visits to support surveillance programs and provide technical assistance. With such guidance, many countries increased the number of specimens tested and reported to FluNet; countries that were partnered with the Influenza Division doubled the annual number of specimens tested for influenza viruses and reported to FluNet from 2013 (>500,000 specimens/year) to 2019 (>1 million specimens/year) (*5*). This testing and sentinel surveillance enabled characterization of the seasonality and temporality of influenza and other respiratory viruses, including human coronaviruses, and it equipped countries with the tools to detect disease clusters and community transmission. MOH in several countries subsequently established non–sentinel-based surveillance systems for respiratory viruses, with support from WHO, the Food and Agriculture Organization of the United Nations, CDC, and international NGOs. Thailand and Bangladesh, for example, established event-based surveillance (*7*) for unusual respiratory events in humans, and Bangladesh, Laos, Vietnam, China, and Kenya established zoonotic surveillance in live bird markets for earlier detection of novel respiratory viruses.

As SARS-CoV-2 spread globally, countries used their SARI/ILI sentinel surveillance systems and case definitions to collect and report case data for COVID-19 surveillance. They also used their non–sentinel-based surveillance systems, including event-based surveillance, to further help identify and track COVID-19 community clusters. This capacity to leverage influenza sentinel surveillance systems for COVID-19 was bolstered by the GISRS platform (*8*) and guidance documents that resulted from the WHO Consultations in March and October 2020 focused on adapting influenza sentinel surveillance systems to include COVID-19 (*9*,*10*). Staff from MOH, national health institutes, the European Centre for Disease Prevention and Control, the Influenza Division, and other expert groups participated in these consultations. They contributed to guidance on monitoring COVID-19 through existing influenza sentinel surveillance systems while maintaining influenza surveillance, adapting algorithms to test for both respiratory viruses, and reporting weekly aggregated sentinel surveillance data through the GISRS platform. WHO and the Influenza Division later held trainings on implementing this guidance (*11*). Countries began reporting their SARS-CoV-2 testing data captured through influenza sentinel surveillance to FluNet, which was made publicly available by WHO region (*12*).

A survey administered by CDC found that by May 2020, 82% of Influenza Division partner countries were using their influenza surveillance systems to identify suspected COVID-19 cases and test for SARS-CoV-2. For example, several countries in Africa with established SARI/ILI surveillance platforms reported using these systems to test for SARS-CoV-2, including Togo, which reported that all its COVID-19–related surveillance activities were conducted using its influenza framework (SARI/ILI sentinel surveillance and routine respiratory disease surveillance systems). Both Mozambique and Nepal used SARI/ILI sentinel surveillance systems to monitor for suspected cases of COVID-19 among persons with a known epidemiologic link or travel history. Jamaica optimized its ILI surveillance to detect suspected COVID-19 clusters as possible signals of community transmission. During the early months of the pandemic, however, MOH mounted national responses with support from WHO and local NGOs and academic and health institutes, in some cases with limited guidance and reagent and protocol distribution from CDC, who was managing the domestic response in the United States. At this stage of the pandemic, the Influenza Division was able to provide the most direct technical assistance to countries supported by field staff. We have summarized milestones and accomplishments of 7 countries where Influenza Division field staff supported local partners in leveraging influenza platforms and integrating SARS-CoV-2 surveillance (*13*–*16*) (Table 1).

**Table 1:** Milestones and achievements of 7 countries that worked closely with CDC Influenza Division field staff as they integrated SARS-CoV-2 and influenza surveillance*

Date	Milestone or achievement
December 2019	• The WHO country office in China learns of cases of viral pneumonia in the city of Wuhan, China, and notifies the International Health Regulations focal point in the WHO Western Pacific Regional Office on December 31, 2019 (*4*).
January 2020	• Vietnam leverages laboratory capacity built for influenza surveillance to begin testing for SARS-CoV-2; it detects the first case in the country on January 23, 2020. • WHO declares that the COVID-19 outbreak is a Public Health Emergency of International Concern on January 30, 2020 (*4*). • India selects 13 of its Virus Research and Diagnostic Laboratories already equipped to conduct influenza virus testing to start testing for SARS-CoV-2 in 11 states (*13*). • South Africa’s NIC at NICD begins testing for SARS-CoV-2 as part of the country’s pneumonia and ILI surveillance system.
February 2020	• Thailand starts testing for SARS-CoV-2 using its influenza platform, including its sentinel surveillance systems.
March 2020	• WHO makes the assessment that COVID-19 is a pandemic on March 11, 2020 (*4*). • Laos starts testing for SARS-CoV-2 through its SARI/ILI sentinel surveillance systems. • Bangladesh starts testing for SARS-CoV-2 through its SARI/ILI sentinel surveillance systems. • NICD (South Africa) confirms the country’s first case of COVID-19 on March 5, 2020; testing is expanded from the NIC at NICD to the Network for Genomic Surveillance in South Africa, a network of public and private laboratories, academic institutions, and scientists.
April 2020	• Kenya starts testing for SARS-CoV-2 through 8 SARI/ILI sentinel surveillance sites in the country.
July 2020	• Vietnam’s severe viral pneumonia surveillance system detects cases from a nosocomial outbreak that leads to the country’s second COVID-19 wave (*14*).
December 2020	• Bangladesh enrolls 1,986 case-patients from its SARI sentinel surveillance sites from March‒December 2020; 285 (14.3%) are infected with SARS-CoV-2, 175 (8.8%) are infected with influenza virus, and 5 (0.3%) are infected with both respiratory viruses (*15*).
February 2021	• NICD (South Africa) starts receiving requests for SARS-CoV-2 sequencing from other countries in the region and accepts specimens for sequencing from Eswatini, Lesotho, Mauritius, Mozambique, Namibia, and Sudan.
September 2021	• Thailand adds a module of questions to its influenza sentinel surveillance forms to assess influenza and COVID-19 vaccination history in 6 sentinel surveillance sites as part of its Influenza and SARS-CoV-2 Vaccine Effectiveness Network.
November 2021	• On November 24, 2021, NICD and the Network for Genomic Surveillance in South Africa report a new variant of SARS-CoV-2 that was detected from specimens collected on November 14, 2021 in South Africa. • WHO designates B.1.1.529 as Omicron, the fifth variant of concern, on November 26, 2021 (*16*).
March 2022	• Thailand’s Influenza and SARS-CoV-2 Vaccine Effectiveness Network reports that of 2,425 specimens collected and tested, 6 (0.2%) are positive for influenza and 573 (23.6%) are positive for SARS-CoV-2; 426 (74.3%) of these SARS-CoV-2–positive specimens are detected during January‒March 2022, a period when >90% of sequenced viruses are the Omicron variant. • Kenya reports having enrolled 6,822 SARI/ILI case-patients through its sentinel surveillance system during April 2020‒March 2022, of whom 738 (10.8%) test positive for SARS-CoV-2, 628 (9.2%) test positive for influenza, and 63 (0.9%) are coinfected with influenza and SARS-CoV-2.

*CDC, US Centers for Disease Control and Prevention; ILI, influenza-like illness; NIC, National Influenza Centre; NICD, National Institute for Communicable Diseases; SARI, severe acute respiratory infection; WHO, World Health Organization.

## Harnessing of Influenza Laboratory Surveillance Infrastructure to Build SARS-CoV-2 Laboratory Surveillance Capacity

GISRS has built international influenza surveillance laboratory capacity that was instrumental in the response to global infectious disease outbreaks, including the 2009 H1N1 pandemic, 2013 H7N9 outbreak, and COVID-19 pandemic. Influenza Division laboratory teams supported NICs in >120 countries and enhanced laboratory diagnostic capacity through the development of novel assays and proficiency panels, reagent distribution, and technical guidance. WHO and the Influenza Division also worked closely with MOH and other partners, such as the Association of Public Health Laboratories, to support in-depth training to build laboratory capacity and prepare countries to respond to respiratory viruses with epidemic and pandemic potential. In 2017, WHO, the Food and Agriculture Organization of the United Nations, the World Organisation for Animal Health, the Influenza Division, and 150 partners from 12 member states updated global epidemiology and laboratory rapid response trainings for respiratory epidemics; these trainings were held in multiple countries in Asia and the Americas in 2019 before the COVID-19 pandemic. By using these resources, NICs optimized their laboratory capacity to harness influenza diagnostic platforms to test for pandemic- and epidemic-prone respiratory viruses, including Middle East respiratory syndrome coronavirus, respiratory syncytial virus, and more recently, SARS-CoV-2. As a GISRS CC and a participant in WHO’s Pandemic Influenza Preparedness Framework (*17*), the Influenza Division also contributed to global respiratory virus genomic sequencing capacity. Staff worked closely with WHO’s Global Influenza Programme in developing influenza genomic surveillance recommendations and with GISAID (https://www.gisaid.org), an initiative founded on sharing influenza virus sequencing data, to develop critical sequencing informatics tools and train partners on their use. Partner countries built their genomic sequencing capacity with support from WHO, the Influenza Division, academic institutions, and institutes of health. For example, Chile established next-generation sequencing in collaboration with the Pan American Health Organization (PAHO) and the Influenza Division, a program that is now being used as a pilot for establishing next-generation sequencing laboratory and informatics pipelines in NICs globally.

As COVID-19 spread globally, NICs and public health laboratories rapidly mobilized to test respiratory specimens for SARS-CoV-2 by using influenza laboratory infrastructure, which was then expanded to intermediary, subnational laboratories. A small case study at the end of this section highlights SARS-CoV-2 testing capacity in countries working with the Influenza Division. 

As part of its support for the global response to the COVID-19 pandemic, the Influenza Division developed and manufactured a research-only use influenza virus and SARS-CoV-2 (Flu SC2) real-time reverse transcription PCR multiplex assay that enables simultaneous detection of influenza A and B viruses and SARS-CoV-2 (*18*). Influenza Division staff conducted hybrid online and in-person training on this assay to aid users globally, including with partners in the WHO Regional Office for the Eastern Mediterranean and PAHO. The Flu SC2 multiplex assay was distributed globally by the International Reagent Resource (IRR). Originally known as the Influenza Reagent Resource, IRR was established in 2008 by CDC to manufacture, stock, and distribute key reagents and test kits globally and to develop and distribute resources for outbreak responses and the detection of emerging pathogens. Although the program experienced challenges and delays in distributing reagents for SARS-CoV-2 testing during most of 2020, IRR organized global distribution of 1,936 kits of the Flu SC2 multiplex assay to 151 laboratories in 134 countries during October 1, 2020–February 28, 2022, corresponding to >968,000 tests. These assays allowed laboratories to conduct more tests in less time while optimizing the use of important testing materials and facilitating uninterrupted surveillance for both influenza and SARS-CoV-2, even as influenza laboratory staff were reassigned to assist with SARS-CoV-2 testing.

During the COVID-19 pandemic, countries leveraged their influenza platforms and trainings to sequence SARS-CoV-2 and publicly share sequencing data through GISAID. For example, Thailand, which received training on next-generation sequencing from the Influenza Division before the pandemic, received additional support to sequence SARS-CoV-2 and used this platform. Chile used its next-generation sequencing platform to identify novel SARS-CoV-2 variants in the Southern Hemisphere (*19*). CDC also received and sequenced SARS-CoV-2 specimens collected globally using the same staff and infrastructure that routinely monitor influenza viruses for antigenic drift. Laboratory and informatics staff from the Influenza Division comprised ≈75% of CDC’s COVID-19 Strain Surveillance and Emerging Variant Team that tracks, sequences, isolates, and antigenically characterizes SARS-CoV-2 variants. Division laboratory staff also developed and performed assays to measure neutralizing activities of sera from SARS-CoV-2–infected or COVID-19 vaccinated persons. These activities helped identify the emergence and spread of SARS-CoV-2 variants of concern and assess correlates of immune protection after natural infection or vaccination. Such activities are anticipated to help with strain selection for future COVID-19 vaccines.

### Case Study 

Using data extracted from Our World in Data (*20*) and Johns Hopkins University (*21*), we described SARS-CoV-2 testing in low- and middle-income countries (LMICs) to evaluate whether those partnered with the Influenza Division were well-positioned to conduct testing and report data. These data were collected from official publicly available sources, usually published by ministries of health or other government entities (*20*,*21*). Partner countries were defined as LMICs that received CDC funding to support influenza surveillance activities since 2013; we identified 64 partner countries. LMICs were considered to have regular testing data if they reported SARS-CoV-2 testing data (inclusive of reverse transcription PCR and antigen tests) on ≥13% of the days that they reported any COVID-19 data (e.g., confirmed cases and hospitalizations). We selected 13% as a cutoff to approximate weekly (4 times in 30 days) reporting to increase comparability between countries.

Of the 64 LMICs partnered with ID, 41 (64%) regularly reported SARS-CoV-2 testing data by June 2020, with >40 million tests (Table 2). By September 2020, 42 partner LMICs (66%) reported >158 million tests, and by October 2021, 45 (70%) reported >1 billion tests. The scale-up in testing capacity in these countries was accomplished despite major shortages in testing reagents and delays in reagent distribution (*22*). Median tests per 1,000 persons were highest during January 2020–October 2021 at 240.7 tests/1,000 persons (interquartile range 90.1–424.8 tests/1,000 persons). Median tests per confirmed COVID-19 case were highest during the January 2020–June 2020 start of the pandemic, at 20.9 tests/confirmed case (interquartile range 9.3–34.4 tests/confirmed case).

**Table 2:** Number of SARS-CoV-2 tests and median number of tests per 1,000 persons and per confirmed COVID-19 case among 64 CDC Influenza Division partner LMICs across 3 periods*

Period	No. (%)	Cumulative no. tests†	Median no. tests/1,000 persons (IQR)†	Median no. tests/confirmed case (IQR)†
Jan–Jun 2020	41 (64)	40,092,751	8.2 (3.6–24.6)	20.9 (9.3–34.4)
Jan–Sep 2020	42 (66)	158,319,895	28.4 (11.8–70.1)	11.6 (6.8–24.2)
Jan 2020–Oct 2021	45 (70)	1,051,798,691	240.7 (90.1–424.8)	8.5 (5.7–14.0)

*Partner countries were defined as LMICs that received CDC funding to support influenza surveillance activities since 2013. LMICs were included if they reported SARS-CoV-2 testing data on >13% of the days that they reported any COVID-19 data (e.g., confirmed cases and hospitalizations) to approximate 4-times-per-month (4/30 days) reporting. CDC, US Centers for Disease Control and Prevention; IQR, interquartile range; LMICs, low- and middle-income countries. †Testing data were extracted from ministry of health and other government webpages, and included either reverse transcription PCR tests, antigen tests, or both.

## Use of Influenza Pandemic Preparedness Plans and Trainings for the COVID-19 Response

For years, countries developed pandemic preparedness plans for their national responses (*23*–*25*) and participated in tabletop and simulation exercises on unusual respiratory events and influenza pandemics with WHO and Influenza Division guidance and training. In November 2019, just before the start of the COVID-19 pandemic, Myanmar and the Association of Southeast Asian Nations led a joint tabletop pandemic response exercise with Laos, Cambodia, WHO, and CDC. During 2018–2019, WHO led a multiregional effort to review National Influenza Pandemic Preparedness Plans (NIPPPs) with support from CDC; officials at the WHO Regional Office for the Eastern Mediterranean (*26*) and PAHO held workshops. These pandemic preparedness activities and exercises facilitated cross-sectoral collaboration between healthcare providers, national reference laboratories, emergency operation centers, and pandemic vaccine deployers during the COVID-19 pandemic. Countries were able to use their NIPPPs to quickly develop and operationalize their COVID-19 strategic preparedness and response plans in the face of this new disease and a rapidly evolving epidemiologic climate. In some cases, the national deployment and vaccination plans developed by countries participating in COVAX (*27*), a program co-led by WHO, the Coalition for Epidemic Preparedness Innovations, and Gavi, the Vaccine Alliance, aimed at ensuring equitable COVID-19 vaccine distribution, were adapted from existing approved NIPPPs.

## Expansion of Existing Influenza Evaluation Projects to Include COVID-19 Program Evaluations and Studies

During the past decade, MOH engaged in research to better understand influenza virus transmission, epidemic timing, disease and economic burden, and influenza vaccine effectiveness and cost-effectiveness in collaboration with the Influenza Division, other CDC divisions, WHO, and academic research groups. With the global spread of COVID-19, these partnerships were leveraged to collect data about SARS-CoV-2 transmission dynamics and, later, COVID-19 vaccine effectiveness. Research sites in Guatemala, India (*28*), Kenya, Peru, South Africa, and Thailand with CDC staff or collaborating with the agency expanded their influenza evaluation portfolios to engage in COVID-19 projects. Influenza population-based surveillance and Influenza Division–supported cohort studies in special populations are being used to investigate laboratory-confirmed SARS-CoV-2 incidence, infection risk and mitigating factors, reinfection, and post–COVID-19 conditions among agricultural workers in Guatemala (*29*), pregnant women in Kenya (*30*), older adults in India, and healthcare providers in Peru (*31*). Several of these cohorts are also examining COVID-19 vaccine effectiveness to SARS-CoV-2 variants by dosing schedules. In Thailand, the Ministry of Health and Influenza Division field staff leveraged close partnerships with academic institutions and hospitals to conduct a serosurvey among health care personnel 1 year after the start of the pandemic (*32*) and after COVID-19 vaccination (*33*). The 13-country PAHO Network for the Evaluation of Vaccine Effectiveness in Latin America and the Caribbean, known as REVELAC-I for its acronym in Spanish (*34*), was activated to assess COVID-19 vaccine effectiveness among hospitalized persons in countries such as Paraguay (*35*); CDC supported this work with financial and technical resources.

## Leveraging of Influenza Vaccine Partnerships for COVID-19 Vaccine Introduction

Influenza vaccine programs are important for pandemic preparedness (*36*) and helped countries prepare for COVID-19 vaccine introduction. An analysis of the 2009 H1N1 pandemic response, for example, found that countries with influenza vaccination programs before the pandemic were more readily able to receive, distribute, and deliver pandemic influenza vaccines (*36*). Efforts for sustainable, seasonal national vaccination programs have been supported by the Partnership for Influenza Vaccine Introduction (PIVI), which includes the Task Force for Global Health, MOH, the Influenza Division, and other groups (*37*). This partnership has provided technical support to MOH in 17 countries and has enabled the distribution of >4.2 million doses of influenza vaccine since 2013 (*38*). During the COVID-19 pandemic, PIVI partnered with CDC’s Global Immunization Division, Center for Global Health, to establish the COVID-19 Implementation Program (CoVIP), whose goal is to support low- and middle-income countries as they administer and evaluate COVID-19 vaccines.

As WHO, MOH, and other international agencies and organizations worked to increase global readiness to implement and evaluate COVID-19 vaccination programs, CoVIP helped >30 partner countries develop workplans to prepare for COVID-19 vaccine rollout and funded all as of August 2021. As part of these activities, the Albania Institute of Public Health and the Armenia National Center for Disease Control used their detailed influenza vaccine distribution microplans for target groups to quickly develop detailed plans for COVID-19 vaccine deployment. CoVIP activities to support partner countries include assistance with safety monitoring, increasing public demand, risk communication, workforce development, data management, and post-introduction and vaccine effectiveness evaluations. Last, PIVI developed a learning agenda to evaluate how existing influenza vaccination platforms for health workers may have facilitated COVID-19 vaccine rollouts.

## Conclusion

The epidemiologic and virologic surveillance systems and programs built for influenza during the past 70 years by MOH, WHO, CDC, and many other partners have been critical to the global response to the COVID-19 pandemic. This report based its influenza and COVID-19 programmatic findings on careful review of peer-reviewed publications, publicly available testing data, archival records of timelines, and CDC records to present the value and importance of investments in influenza surveillance and programs for the COVID-19 pandemic response. However, this report is limited because it focuses on CDC’s international influenza program through the Influenza Division and its role as partners responded to the COVID-19 pandemic but does not exhaustively cover the work and achievements of other influenza program stakeholders. We do not have comprehensive information about MOH, WHO, NGOs, local academic and health institutes, and funding organizations’ COVID-19 pandemic response investments and thus were unable to systematically describe and incorporate their contributions during the pandemic.

As the world adjusts to a long-term strategy for COVID-19 mitigation, the integration of COVID-19 surveillance into existing influenza sentinel surveillance systems and GISRS will facilitate continued global surveillance for respiratory viruses with epidemic and pandemic potential. Staff from MOH, national health institutes, the Influenza Division, and other expert groups contributed to WHO’s recent revised interim guidance, End-to-End Integration of SARS-CoV-2 and Influenza Sentinel Surveillance, published in January 2022 (*39*). CDC’s Influenza Division will continue to support its partner countries as they implement this end-to-end integration and monitor trends and seasonality of SARS-CoV-2 and influenza viruses through cooperative agreements with countries and WHO Regional Offices, laboratory capacity building efforts, and reagent distribution through IRR. Influenza Division staff are working with WHO to revise SARI/ILI sentinel surveillance assessment tools to better document and strengthen countries’ capacity to monitor SARS-CoV-2 and influenza viruses through existing sentinel sites and national programs. IRR continues to distribute the Flu SC2 multiplex assay globally, and the Influenza Division is working with NICs to develop next-generation sequencing workflows to characterize influenza A, influenza B, and SARS-CoV-2 specimens through timely quality sequencing of representative viruses. Influenza Division laboratory staff and the Association of Public Health Laboratories are working with WHO Regional Offices to conduct trainings with NIC and national influenza laboratory staff on the Flu SC2 multiplex assay and influenza and SARS-CoV-2 next-generation sequencing molecular and informatic pipelines. Finally, the vaccine effectiveness evaluations and epidemiologic investigations that the Influenza Division supports through partnerships with WHO Regional Offices, MOH, academic institutions, Task Force for Global Health, CDC field offices, and other in-country collaborators will continue to build upon enhanced surveillance and genomic sequencing pipelines to help assess COVID-19 vaccine effectiveness to emerging SARS-CoV-2 variants, which MOH can use to help develop national COVID-19 vaccination programs and boosting schedules.
